# GUS Reporter-Aided Promoter Deletion Analysis of *A. thaliana POLYAMINE OXIDASE 3*

**DOI:** 10.3390/ijms24032317

**Published:** 2023-01-24

**Authors:** Varvara Podia, Dimitris Chatzopoulos, Dimitra Milioni, Dimitrios J. Stravopodis, Irene Dervisi, Andreas Roussis, Kalliopi A. Roubelakis-Angelakis, Kosmas Haralampidis

**Affiliations:** 1 Section of Botany, Biology Department, National and Kapodistrian University of Athens, 15784 Athens, Greece; 2 Section of Cell Biology and Biophysics, Biology Department, National and Kapodistrian University of Athens, 15784 Athens, Greece; 3 Biotechnology Department, Agricultural University of Athens, 11855 Athens, Greece; 4 Department of Biology, University of Crete, 70013 Heraklion, Greece

**Keywords:** abiotic stress, abscisic acid, *Arabidopsis thaliana*, GUS, jasmonic acid, PAO, polyamines, promoter deletion analysis, salicylic acid, TFBS

## Abstract

Polyamine oxidases (PAOs) have been correlated with numerous physiological and developmental processes, as well as responses to biotic and abiotic stress conditions. Their transcriptional regulation is driven by signals generated by various developmental and environmental cues, including phytohormones. However, the inductive mechanism(s) of the corresponding genes remains elusive. Out of the five previously characterized Arabidopsis *PAO* genes, none of their regulatory sequences have been analyzed to date. In this study, a GUS reporter-aided promoter deletion approach was used to investigate the transcriptional regulation of *AtPAO3* during normal growth and development as well as under various inductive environments. *AtPAO3* contains an upstream open reading frame (uORF) and a short inter-cistronic sequence, while the integrity of both appears to be crucial for the proper regulation of gene expression. The full-length promoter contains several *cis*-acting elements that regulate the tissue-specific expression of *AtPAO3* during normal growth and development. Furthermore, a number of TFBS that are involved in gene induction under various abiotic stress conditions display an additive effect on gene expression. Taken together, our data indicate that the transcription of *AtPAO3* is regulated by multiple environmental factors, which probably work alongside hormonal signals and shed light on the fine-tuning mechanisms of *PAO* regulation.

## 1. Introduction

Polyamines (PAs) play significant roles in all organisms, including plants, both during normal growth and development, and in response to various endogenous and exogenous stresses. Polyamine oxidases (PAOs) use flavin adenine dinucleotide (FAD) as a cofactor to catabolize the PAs spermine (Spm) and spermidine (Spd) with two distinctive biochemical pathways. The back conversion pathway type (BC-type) concerns the catalysis of Spm to Spd and subsequently to putrescine (Put) with the simultaneous production of 3-aminopropanal and hydrogen peroxide (H_2_O_2_) [1,2]. On the other hand, the terminal conversion pathway (TC-type) refers to the terminal oxidation of Spm and Spd to N-(3-aminopropyl)-4-aminobutanal and 4-aminobutanal respectively, as well as 1,3-diaminopropane and H_2_O_2_ [3,4]. The generated H_2_O_2_ acts as a signal for the regulation of effector-gene expression and/or induction of programmed cell death (PCD) syndrome, depending on its size and rate of production [5,6].

The *Arabidopsis thaliana* genome contains five *PAO* genes, *AtPAO1* to *AtPAO5*, which catalyze BC-type reactions. AtPAO1 and AtPAO5 proteins are cytoplasmic, while the other three are peroxisomal. The most studied *A. thaliana PAO* gene is *AtPAO5*, which is expressed in all developmental stages of Arabidopsis, with stronger expression in mature leaves, vascular tissues, flowers, and stems. *AtPAO5* catalyzes the back conversion of Spm and T-Spm to Spd, and has been correlated with xylem differentiation, stem elongation, stress tolerance, and the development of rosette leaves and veins [7,8,9,10]. *AtPAO1*, which prefers Spm, T-Spm, and Nor-Spm as substrates, is expressed in the root transition region and anthers and is involved in stress tolerance, root development, and fertility [11,12,13,14]. *AtPAO2* utilizes Spd, Spm, and T-Spm as substrates, while its expression has been confirmed in root and shoot meristems, anthers, and the main vein of rosette leaves [13,14,15]. *AtPAO4*, which converts predominantly Spm and T-Spm to Spd, is induced during all developmental stages of Arabidopsis, with stronger expression in root and floral organs. Its function has been correlated with various plant processes, such as delayed dark-induced senescence, root development, and fertility [13,14,16,17].

AtPAO3 displays an 84% amino acid identity with AtPAO2, 61% with AtPAO4, and very low similarity to the mammalian PAOs. Although it prefers Spd as its substrate, AtPAO3 oxidizes both Spm to Spd and Spd to Put, while it is inhibited by guazatine [18]. Under normal growth conditions, *AtPAO3* is expressed in two- to five-day-old seedlings in the lateral root cup and columella cells, the elongation and differentiation zones of the roots up to the hypocotyl–root junction site. Its expression is evident in the epidermis, the cortex, the pericycle, and the vascular system. In five- to eight-day-old seedlings, *AtPAO3* expression is apparent in stipules, trichomes, and guard cells [14]. During reproductive development, the gene is expressed in very young flower buds and later, during flower opening, in the walls and the septum of pistils and filaments, as well as in anthers and pollen grains. Pollen staining persists during pollination and pollen tube growth in the nectars and guard cells of the sepals [14]. The activity of *AtPAO3* was also correlated with the elongation of the pollen tube [19], the T-Spm catabolism, and the resistance of *Arabidopsis thaliana* to *Pseudomonas viridiflava* [20]. AtPAO3 is localized to peroxisomes due to its peroxisomal targeting signal, which resides at the C-terminal end of the protein. Given the involvement of peroxisomes in stress responses, *AtPAO3* mRNA has been found to accumulate under normal conditions and various environmental stresses. ABA and wounding induced *AtPAO3* gene expression and mRNA accumulation 1 h post-treatment, while JA, SA, and flagellin-22-treated seedlings showed similar mRNA accumulation profiles after 6 h [5].

Despite the important role of *PAO* genes in a variety of plant developmental processes and abiotic stress responses, little is known concerning their transcriptional regulation and the involvement of putative transcription factor binding sites (TFBS) in gene induction. Here, we generated several chimeric genes, composed of a series of deletions of the *AtPAO3* regulatory sequences and the β-glucuronidase gene (*GUS*), to investigate the *cis*-regulatory elements located upstream of the *PAO3* ORF. Qualitative and quantitative analyses of transgenic *A. thaliana* plants revealed that *AtPAO3* displayed a tissue-specific expression pattern during normal plant growth and development, while several putative TFBS are necessary to induce the gene under various abiotic stress conditions and hormonal treatments. To the best of our knowledge, this is the first comprehensive analysis of a *PAO* gene regulatory sequence. The data obtained from the present study elucidate the importance of specific *cis*-elements in *AtPAO3* gene expression. This understanding is important and may contribute to the contextual exploitation of the putative TFBS in future plant improvement strategies.

## 2. Results

### 2.1. In Silico Analysis of the AtPAO3 Regulatory Sequence

**Figure 1:**
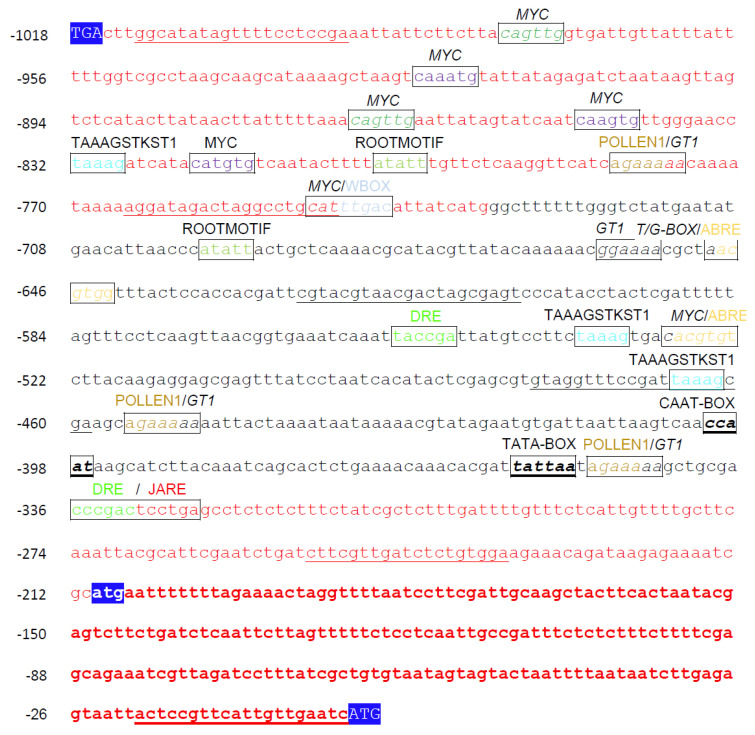
Sequence of the 5’ −regulatory region of the *AtPAO3* gene (*At*3g59050). The most prevalent putative transcription factor binding sites (TFBS) are highlighted. See relevant text for details. Sequence marked in red corresponds to the annotated 3’−end of the previous intergenic area in the order of the chromosome (*At*3g59040) and the 5’−UTR of the *AtPAO3* gene. Sequence in bold red letters represents the ORF upstream (uORF) of the *AtPAO3* coding region (mORF). The underlined sequences indicate the regions used to design the primers for the promoter deletion constructs.

To investigate the regulation of *AtPAO3* gene expression, we initially conducted an in-depth in silico analysis of the *AtPAO3* promoter sequence. The analysis revealed the presence of multiple putative *cis*-regulatory elements within the promoter, which likely regulate the tissue-specific expression of *AtPAO3* during normal growth and development, as well as under various biotic and abiotic stress conditions (Figure 1 and Table S2). Apart from the predicted core promoter elements, such as the TATA- and the CAAT-box, which are located at positions −352 and −397 bp, respectively (upstream of the translation initiation codon), two putative tissue-specific elements and a cell type-specific element were also identified. These were the POLLEN1LELAT52 (AGAAA) element at positions −346, −451, and −778 bp, which is associated with the expression of genes during anther and pollen development [21], the root-specific expression element ROOTMOTIFTAPOX1 (ATATT), located at positions −692 and −800 bp [22] and the guard cell-related element TAAAGSTKST1 (TAAAG) at −828, −533, and −462 bp [23]. The regulatory sequence of *AtPAO3* also contains a number of presumed elements related to the expression of the gene at specific developmental stages and responses under diverse environmental or adverse conditions. Among them are the ABRE elements (ABA-responsive elements, ACGTG) located at positions −523 and −643 bp [24,25,26], and the salt-induced GT1GMSCAM4 (GAAAAA) elements [27] at positions −344, −449, and −776 bp. Furthermore, it includes several putative TFBS, which have been associated with dehydration and osmotic stress [28,29]. These include MYC transcription factor binding sites (MYCCONSENSUSAT, CANNTG) at positions −526, −743, −816, −842, −863, −919, and −973 bp, as well as two dehydration responsive elements (DRE) at positions −549 and −331 bp, which consist of the consensus sequence A/GCCGAC/G or TACCGACAT [30]. Another category of TFBS located within the *AtPAO3* promoter sequence includes the phytohormone-responsive elements. The analysis revealed the presence of a GT1CONSENSUS (GRWAAW) at positions −344, −449, −654, and −776 bp [31], and a WBOXATNPR1 (TTGAC) at −741 bp [32], which have been characterized as salicylic acid (SA) responsive elements. Additionally, two jasmonic acid (JA)-responsive elements, the T/GBOXATPIN2 (AACGTG) and JARE (G(C)TCCTGA) are also present at positions −644 and −325 bp, respectively [33].

### 2.2. Analysis of the Regulatory Sequences of AtPAO3

**Figure 2:**
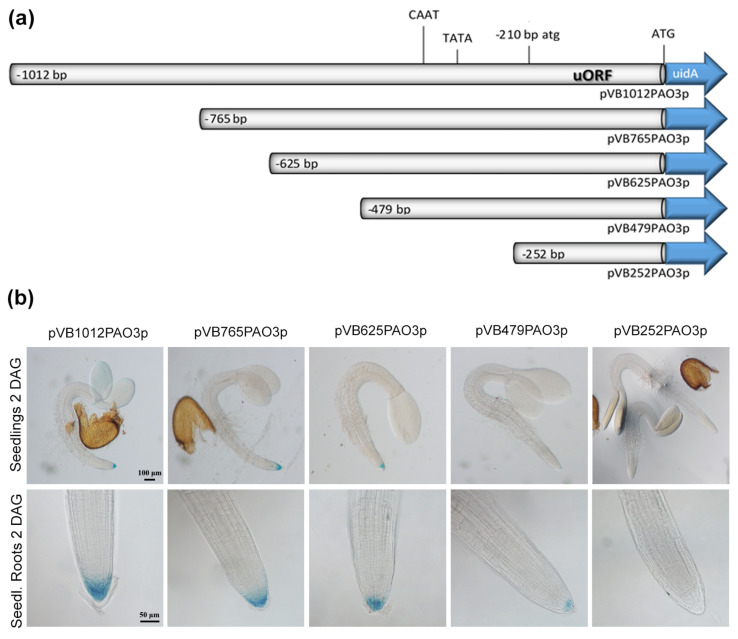
(**a**) Graphic presentation of the *AtPAO3* regulatory sequences fused to the GUS reporter gene to generate the deletion constructs pV1012PAO3p, pVB765PAO3p, pVB625PAO3p, pVB479PAO3p, and pVB252PAO3p. The atg and ATG translational start codons of the uORF and the uidA ORF, respectively, as well as the predicted TATA−box and CAAT−box are indicated. (**b**) Representative images of GUS−stained 2− to 3−day−old seedlings and root tips, under normal growth condition, showing the decrease in GUS expression with the progressive shortening of the promoter length. Note that in construct pVB252PAO3p, which contains only the uORF and 42 bp of the *AtPAO3* promoter, GUS expression was non−detectable.

The *AtPAO2* gene has been shown to contain an uORF, which negatively affects GUS expression in a polyamine-dependent manner [34]. The *AtPAO3* gene locus also contains an uORF, whose canonical start codon (atg) is located 210 bp upstream of the PAO3 mORF start codon (ATG), encoding for a small upstream peptide of 68 amino acids. The intercistronic region between the uORF and the main coding region of *AtPAO3* consists of only the three nucleotides ATC. It has been shown that the expression of the uORF typically interferes with the expression of a downstream ORF, whereas increasing the intercistronic spacing by inserting additional sequences reduces the inhibitory effect of the uORF [35]. In order to evaluate the contribution of the uORF and the identified regulatory *cis*-acting elements to *AtPAO3* expression, we generated a series of transgenic Arabidopsis lines. The full regulatory sequence, as well successive deletions of the *AtPAO3* promoter were translationally fused to the β-glucuronidase (*GUS*) reporter gene, and the generated constructs were introduced into *A. thaliana* plants (Figure 2a). At least 10–12 independent transgenic lines for each construct were qualitatively and quantitatively analyzed for GUS expression during normal vegetative and reproductive development and under various environmental stress conditions.

**Figure 3:**
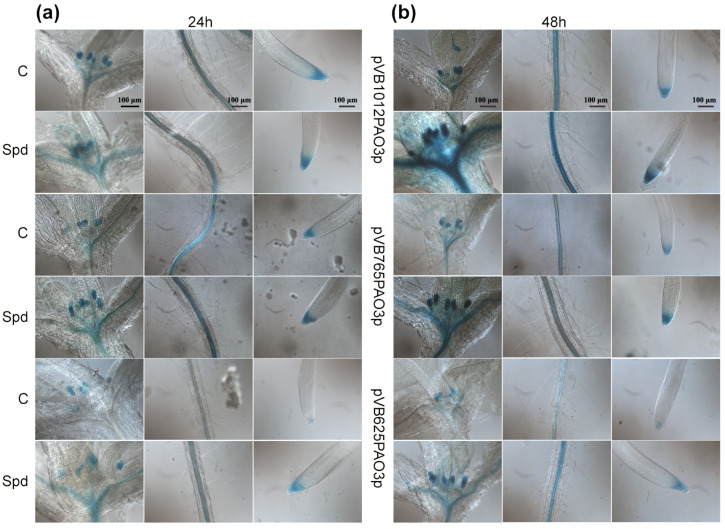
Response of GUS lines to Spd treatment. Representative images of GUS−stained transgenic Arabidopsis lines harboring the constructs pVB1012PAO3p, pVB765PAO3p, and pVB625PAO3p at normal conditions (C), and after Spd treatment. Images of shoot (left panel), root elongation and differentiation zone (middle panel), and root tip (right panel), before and after Spd treatment, for 24 h (**a**) and 48 h (**b**).

To verify whether the *AtPAO3* promoter deletion constructs function correctly, representative transgenic lines were subjected to Spd treatment [13]. As shown in Figure 3, the full promoter (pVB1012PAO3p) displayed, at normal conditions, a characteristic tissue-specific expression pattern with strong GUS staining in the root tip, the vascular cylinder, and the leaf primordia. In response to Spd treatment, a significant increase in GUS expression was evident after 24 h and 48 h in all tissues. While the gradual deletion of the promoter resulted in a decrease in gene expression under normal conditions, all transgenic plants displayed elevated GUS staining after 24 h or 48 h of Spd administration, indicating that the transcriptional fusion constructs responded properly to the treatment with Spd (Figure 3). However, mutagenizing the intercistronic ATC nucleotides (spacer sequence) to CC resulted in a complete loss of gene expression, either under normal or stress conditions (data not shown). The latter suggests that both the presence of the uORF and the spacer sequence are necessary for proper *AtPAO3* expression, while compromising the integrity of the ATC intercistronic region abolishes completely the ability of the promoter to regulate *AtPAO3* mORF expression.

### 2.3. Assessment of the Putative TFBS during Normal Growth and Development

**Figure 4:**
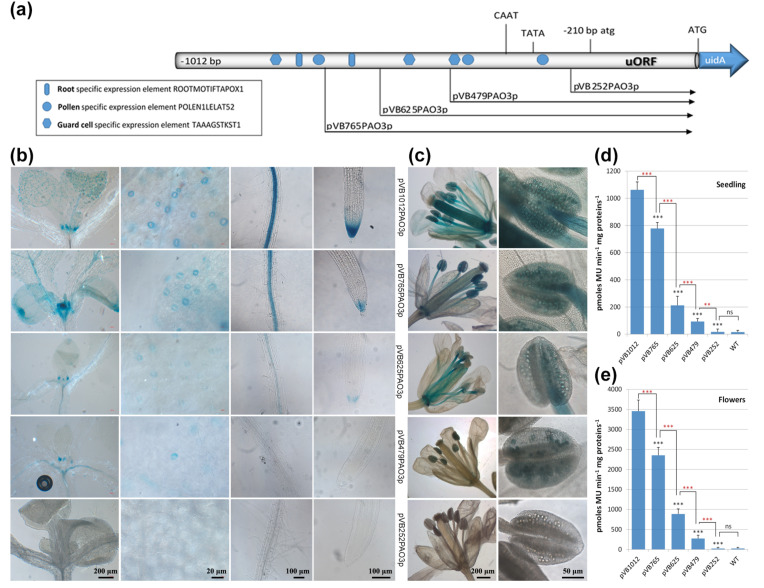
GUS−assisted *PAO3* expression pattern during normal vegetative and reproductive development. (**a**) Graphic presentation of the deletion construct and the location of the major relevant TFBS within the promoter sequence. The atg and ATG translational start codons of the uORF and the uidA ORF, respectively, as well as the predicted TATA−box and CAAT−box are indicated. Representative images showing the GUS staining pattern in 8−day−old seedlings (**b**) and flower tissues (**c**) in relation to promoter size. Quantitative GUS expression measurements in whole seedlings (**d**) and flowers (**e**). Non−transgenic wild−type (WT) Arabidopsis plants were used as control for monitoring non−specific background of GUS activity. Data are means ± SE of three biological replicates (*n* = 12). Statistical analysis was performed using one−way analysis of variance (ANOVA), followed by Tukey’s test for multiple comparisons. Black asterisks denote significant differences between each deletion construct compared to the full promoter, while red asterisks denote significant differences between the deletion lines (*** or *** *p* < 0.001, ** *p* < 0.01, ns: non-significant).

In 2- to 3-day-old seedlings, plant lines harboring the full-length promoter transgene construct (pVB1012PAO3p) showed high expression of the reporter gene in the lateral root cap and columella cells, and moderate expression in the hypocotyl and the cotyledons (Figure 2b). Successive deletion of the promoter to point −479 bp (pVB479PAO3p) gradually restricted GUS expression only to the columella cells, while deletion of the promoter to point −252 bp (pVB252PAO3p) completely abolished the expression of the reporter gene in all tissues. Construct pVB252PAO3p harbors only the uORF and 42 bp of the promoter sequence (Figure 2a). In 8-day-old seedlings, GUS expression was evident in the root tip, in the vascular tissue of the root elongation and differentiation zone up to the hypocotyl–root junction site, in the primordia of newly expanding leaves, and in guard cells (Figure 4b). Deletion of the promoter to point −765 bp (construct pVB765PAO3p) resulted in a decrease in GUS expression in roots (vascular cylinder and root cap), while the expression in the aerial shoot tissues seemed to be unaffected. This is probably due to the deletion of the putative root-specific element, located at position −800 bp. However, deletion of the promoter to point −625 bp resulted in a significant decrease in GUS staining in all tissues. Further deletion of the promoter to point −479 bp proved to affect predominantly the roots, which exhibited a very faint GUS signal, compared to the less affected expression in the shoot tissues. Similarly, GUS expression is prominent in guard cells of transgenic plants harboring constructs pVB1012PAO3p and pVB765PAO3p. Further deletion of the promoter gradually decreased gene expression, which, however, was still evident in construct pVB479PAO3p harboring the most proximal guard cell-specific expression element. As expected, GUS expression was completely abolished in all tissues of the pVB252PAO3p transgenic line, harboring only the uORF and 42 bp of the promoter sequence (Figure 4b). During reproductive development, GUS expression, driven by the full promoter, was prominent during flower opening in pistils, pollen grains, and the anther filaments. In pollen, GUS expression persists also during pollination (data not shown). The progressive deletion of the promoter to point −479 bp led to a gradual decrease in reporter gene expression in all flower tissues. GUS staining was only evident in the pollen grains of anthers, probably due to the presence of the two pollen-specific elements located at positions −346 bp and −451. Reporter gene expression was again eliminated in construct pVB252PAO3p, which harbors only 42 bp of the promoter and the uORF sequence (Figure 4c). Quantitative GUS measurements performed on 8-day-old seedlings and flower tissues were in line with the expression pattern described above (Figure 4d,e).

### 2.4. Heat Stress Does Not Affect AtPAO3 Expression

Temperature fluctuations are among the most severe environmental stresses faced by plants. The in silico analysis did not identify any canonical or deviated heat shock element (HSE) [36], within the *AtPAO3* promoter sequence. However, since *AtPAO3* expression is influenced by ABA [18] and the application of exogenous ABA has been shown to improve the tolerance of crop plants to heat stress [37], we investigated whether *AtPAO3* is induced indirectly under this hazardous condition. None of the transgenic lines tested for heat inducibility showed any increase in reporter gene expression. GUS staining was similar in the aerial parts of the control and heat-shock-treated plants, while in root tips, a slight decrease in gene expression was detected (Figure S1). This is in accordance with microarray data obtained from the Arabidopsis eFP Browsers and results showing that underexpression of *ZmPAO* correlates with increased thermotolerance in tobacco plants, whereas *ZmPAO* overexpressors exhibit significant impairment of thermotolerance [38].

### 2.5. The AtPAO3 Promoter Responds Strongly to Salinity and ABA Treatment

**Figure 5:**
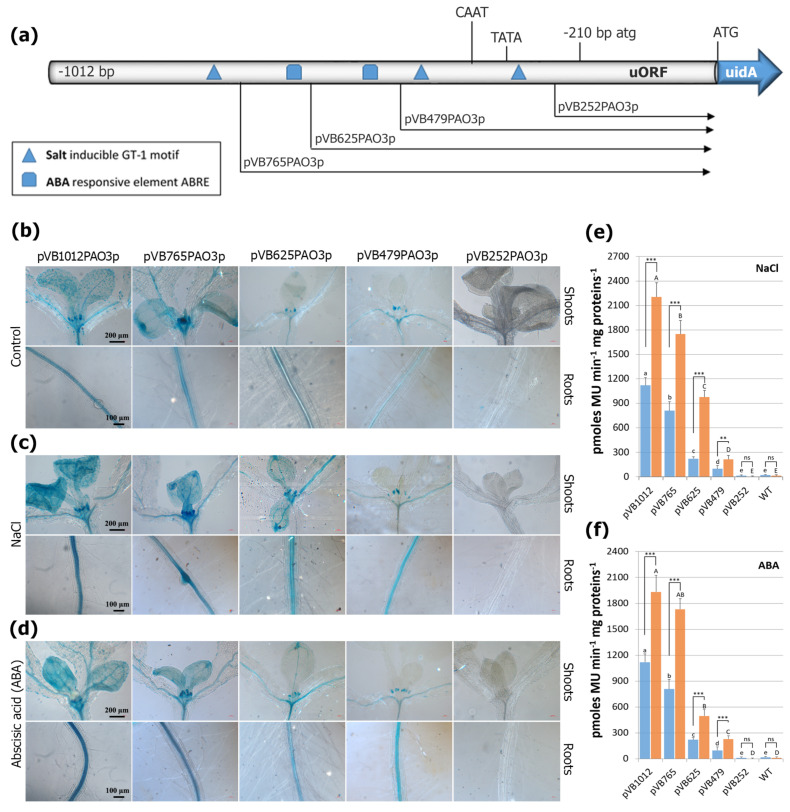
GUS−mediated *PAO3* expression pattern after salinity and ABA treatment. (**a**) Graphic presentation of the deletion constructs and the location of the major relevant TFBS within the promoter sequence. The atg and ATG translational start codons of the uORF and the uidA ORF, respectively, as well as the predicted TATA−box and CAAT−box are indicated. Representative images showing the GUS−staining pattern in 8-day-old transgenic seedlings in control plants (**b**), and after salinity (**c**) and ABA treatment (**d**) in relation to promoter size. (**e**,**f**) Quantitative GUS expression measurements in whole seedlings after salinity (**e**) and ABA treatment (**f**), respectively. Non−transgenic wild−type (WT) Arabidopsis plants were used as control for monitoring non−specific background of GUS activity. Data are means ± SE of three biological replicates (*n* = 12). Statistical analysis was performed using two−way ANOVA followed by Tukey’s test for multiple comparisons. Lowercase and uppercase letters denote significant differences between deletion lines under control conditions (blue bars) and treatments (orange bars), respectively (*p* < 0.05), while asterisks indicate differences between control and treated plants for each deletion line (*** *p* < 0.001, ** *p* < 0.01, ns: non-significant).

Endogenous levels of ABA can promote the accumulation of PAs and are known to play a crucial role in biotic and abiotic stress responses [4], while exogenously applied ABA can regulate growth quality under salt stress [37]. Salinity, on the other hand, induces high Na^+^ content, particularly in leaves, and triggers the production of reactive oxygen species (ROS), such as H_2_O_2_, which mediates the regulation of ABA catabolism [39] and may act as a signaling molecule in several physiological and biochemical processes [40]. Increasing evidence suggests that H_2_O_2_ is involved in the regulation of Na+/K+ homeostasis under salt stress [41]. Given that PAO genes are induced by salinity and their expression has been associated with salt stress tolerance in various plant species [5,8,18,42,43,44,45], we evaluated the *pAtPAO3*::*GUS* transgenic lines for their response to NaCl and ABA treatment. As shown in Figure 5, the full promoter (pVB1012PAO3) displayed increased expression in both treatments. The *PAO3* regulatory sequence contains three putative salt-inducible GT-1 motifs, which are located at various positions within the promoter. By gradually deleting these elements, a gradual and progressive decline in GUS reporter gene expression was observed, while in construct pVB252PAO3, the absence of all three GT-1 TFBS resulted in undetectable levels of GUS staining. In line with the distribution of the putative ABRE was also the response of the promoter to ABA treatment. Constructs pVB1012PAO3 and pVB765PAO3 exhibited similar GUS expression levels, probably due to the lack of an ABA-responsive element in fragment −1012 to −765 bp. However, deleting the promoter to point −479 bp removed the ABRE at position −643 bp, causing a significant decrease in reporter gene expression. By removing the second ABRE, a further decline in GUS staining was observed. The inducibility of construct pVB479PAO3 by ABA was non-significant compared to that of the control plants, which is consistent with the absence of any ABRE in the promoter sequence up to point −479 bp.

### 2.6. Assessment of Putative TFBS after D-Mannitol Treatment

**Figure 6:**
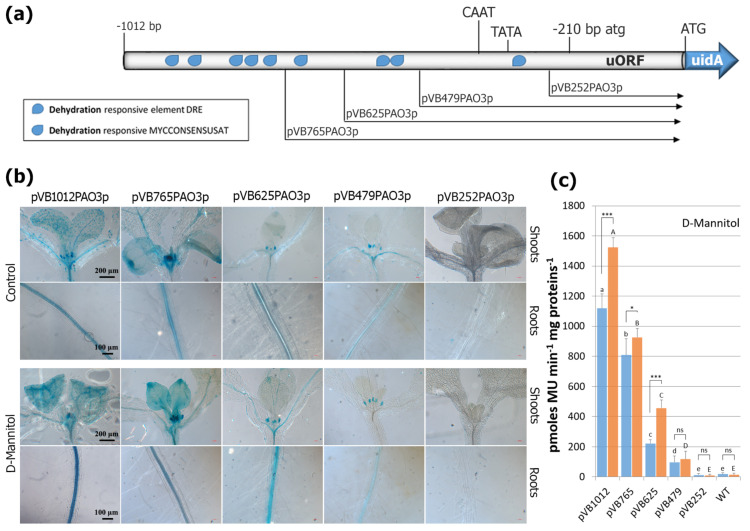
GUS−mediated *AtPAO3* expression pattern after dehydration/osmotic stress induced by D-mannitol. (**a**) Graphic presentation of the deletion constructs and location of the major relevant TFBS within the promoter sequence. The atg and ATG translational start codons of the uORF and the uidA ORF, respectively, as well as the predicted TATA−box and CAAT−box are indicated. (**b**) Representative images showing the GUS−staining pattern in 8−day−old transgenic seedlings in control plants (upper panel) and after D−mannitol treatment (lower panel) in relation to promoter size. (**c**) Quantitative GUS−expression measurements in whole seedlings after D−mannitol treatment. Non−transgenic WT Arabidopsis plants were used as control for monitoring non−specific background of GUS activity. Data are means ± SE of three biological replicates (*n* = 12). Statistical analysis was performed using two−way ANOVA followed by Tukey’s test for multiple comparisons. Lowercase and uppercase letters denote significant differences between deletion lines under control conditions (blue bars) and treatments (orange bars), respectively (*p* < 0.05), while asterisks indicate differences between control and treated plants for each deletion line (*** *p* < 0.001, * *p* < 0.05, ns: non-significant).

Mannitol is a growth-repressive compound that mimics the dehydration-osmotic stress in plants experiencing water scarcity under various environmental conditions. During dehydration, the ABA-mediated closure of stomata is of special importance for lowering transpiration and retaining water within the leaf mesophyll [46]. Given the link between dehydration-osmotic stress and ABA, we assessed the promoter GUS lines for their response to mannitol treatment. As shown in Figure 6, the *AtPAO3* promoter contains several putative dehydration and osmotic stress-responsive elements, such as the DRE (A/GCCGAC/G or TACCGACAT) and MYC (CANNTG) TFBS at various positions. Most of the MYC elements are located far upstream of the ATG translational start codon, while the two DRE elements are located at positions −549 and −331 bp, respectively. The full promoter displayed a moderate elevation of GUS activity after mannitol treatment. By deleting most of the MYC elements located at the 5′ end of the promoter, the remaining regulatory sequences led only to a small increase in reporter gene expression by mannitol. Further deletion of the promoter, and consequently the TFBS located proximal to the mORF start codon, resulted in a sequential decrease in GUS staining, indicating an additive effect of the dehydration and osmotic stress-responsive elements in gene expression. The expression of GUS in the transgenic lines harboring the construct pVB252PAO3 was comparable to that of the control plants and in line with all other treatments, suggesting that the minimal functional *AtPAO3* regulatory sequence has a length of 479 bp (including the uORF).

### 2.7. Evaluation of the AtPAO3 Regulatory Sequences to JA and SA Treatments

**Figure 7:**
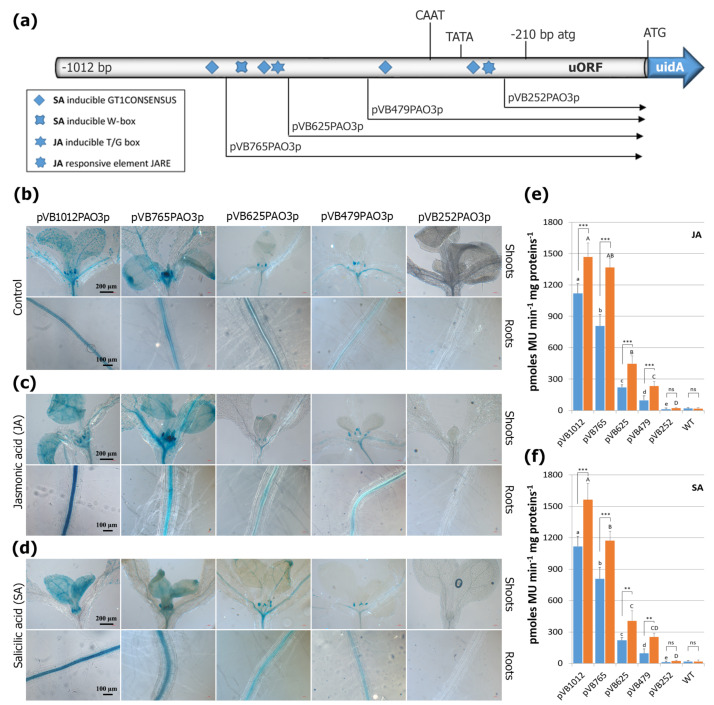
GUS−mediated PAO3 expression pattern after JA and SA treatment. (**a**) Graphic presentation of the deletion constructs and location of the major relevant TFBS within the promoter sequence. The atg and ATG translation start codons of the uORF and the uidA ORF, respectively, as well as the predicted TATA−box and CAAT−box are indicated. Representative images showing the GUS−staining pattern in 8−day−old transgenic seedlings of control plants (**b**), and after JA (**c**) and SA treatment (**d**) in relation to promoter size. (**e**,**f**) Quantitative GUS-expression measurements in whole seedlings after JA (**e**) and SA treatment (**f**), respectively. Non−transgenic wild−type (WT) Arabidopsis plants were used as control for monitoring non−specific background of GUS activity. Data are means ± SE of three biological replicates (*n* = 12). Statistical analysis was performed using two−way ANOVA followed by Tukey’s test. Lowercase and uppercase letters denote significant differences between deletion lines under control conditions (blue bars) and treatments (orange bars), respectively (*p* < 0.05), while asterisks indicate differences between control and treated plants for each deletion line (*** *p* < 0.001, ** *p* < 0.01).

Both JA and SA are important plant growth regulators that have been shown to play key biological functions under various biotic and abiotic stress conditions [47,48]. Moreover, an elaborated signaling crosstalk between ABA, JA, and SA is known to facilitate plants’ integration of different biotic and abiotic stress responses [49,50,51,52]. To evaluate the response of the *AtPAO3* promoter to JA and SA, the transgenic lines harboring the promoter-GUS fusion constructs were subjected to treatment with the above-mentioned hormones. In Figure 7, representative images of GUS-stained tissues and the respective quantitative GUS assays of whole seedlings are shown. As far as JA treatment is concerned, the *AtPAO3* promoter contains only two JA responsive elements, one T/G-box at position −644 and one JARE at position −325. Deletion of the promoter to point −765 bp retains these two TFBS, and hence, the GUS-staining intensity of the lines harboring constructs pVB1012PAO3 and pVB765PAO3 is comparable and in line with the quantitative GUS measurements (Figure 7c,e). As expected, deleting the promoter to point −625 bp resulted in a drastic decline in GUS expression due to the removal of the upstream JA T/G-box element. However, the most proximal JARE element was still able to confer a moderate induction of the reporter gene under JA treatment. Deletion of the promoter to point −479, simply shortens the promoter sequence while retaining the JARE element and the inducibility of construct pVB479PAO3 by JA. This is clearly reflected in the GUS assay, which shows a slight decrease in GUS activity in both untreated and treated plants, compared to construct pVB625PAO3.

As far as the phytohormone SA is concerned, the five *cis*-acting promoter elements (GT1 consensus sequences and W-box elements), which have been associated with the responsiveness to SA, are scattered throughout the *AtPAO3* regulatory sequence. Progressive deletion of the promoter resulted in a decline in GUS expression after SA treatment. Removal of the most distal element, located at position −776, results in a decline in GUS activity. However, the inducibility of this promoter fragment (pVB765PAO3) remained strong. Further deletion of the promoter to point −625, which removes two additional SA responsive elements, resulted in a significant decline of GUS activity. As with JA, the promoter fragment −625 to −479 bp seems to lack any putative SA TFBS. Hence, the respective constructs displayed similar expression levels (Figure 7f). The inducibility of construct pVB479PAO3 by SA was still significant, probably due to the presence of the two proximal SA-responsive elements within this promoter fragment. As expected, deletion of the promoter to point −250 bp eliminated both the JA and SA binding sites, resulting in the nullification of gene expression.

## 3. Discussion

The low molecular weight PAs are positively charged aliphatic polycations, which are involved in diverse cellular processes, such as cell signaling, gene expression, and cell proliferation. As such important molecules, their cellular homeostasis is regulated through mechanisms affecting their biosynthesis, conjugation, compartmentalization, catabolism, and cellular transport [53,54]. The catabolic mechanisms of PAs involve copper-containing amine oxidases (CuAOs) and PAOs, which cause the production of H_2_O_2_. This and other ROS act as signaling molecules with integral roles in growth, development, and responses to biotic and/or abiotic stimuli, conferring systemic acquired resistance (SAR) or systemic acquired acclimation (SAA) in plants [4,6]. Despite the long-lasting characterization of the five *A. thaliana PAO* loci, no comprehensive promoter analysis has been reported for any of the genes to date. *AtPAO3* has previously been implicated in ROS homeostasis [55] and pollen tube growth [19], while the accumulation of *PAO3* mRNAs has been reported after ABA, JA, and SA treatment [18]. In view of its significance in various plant processes, here we provide data from a detailed analysis of the *AtPAO3* promoter and its uORF.

Prokaryotic and eukaryotic gene expression is often regulated by an uORF, which is located within the 5′-UTR of an mRNA. Translation of the uORF typically inhibits downstream expression of the primary ORF (mORF). While, in plants, ca. 24–30% of mRNAs contain uORFs, only a few of them have been functionally characterized so far, and their regulatory mechanisms are poorly understood [35]. This element has been associated with the fine-tuned regulation of genes involved in various developmental processes, signaling, and stress responses [56,57]. The uORF of *AtPAO3* encodes for a small upstream peptide of 68 amino acids, while the intercistronic region consists of the nucleotides ATC. Our results showed that the intercistronic region is crucial for proper inducibility of the reporter gene by Spd. Mutagenesis of the ATC nucleotides resulted in the annulment of GUS expression, suggesting that its integrity is vital for *AtPAO3* induction. This is in line with previous results showing a misregulation of *AtPAO2* in the absence of the respective uORF. Transgenic lines harboring the native *AtPAO2* promoter or the constitutive CaMV 35S promoter fused to the uORF-containing UTR showed prominent translational repression compared with that of the native promoter fused to the UTR without the uORF. Moreover, the *AtPAO2* uORF affected reporter gene expression in a PA-dependent manner. While the transgenic plants displayed an uORF-dependent negative effect on GUS expression, exogenous applications of PAs alleviated this negative effect, resulting in a positive modulation of gene expression [34].

The in silico analysis of the *AtPAO3* promoter revealed the presence of several putative TFBS, which are involved in the regulation of the gene during normal growth and development. After confirming the proper inducibility of the generated constructs by Spd, we examined the developmental expression of *AtPAO3* in seedlings and inflorescence tissues. While in 2 to 3-day-old seedlings expression was confined in the columella cells of the root tip, the cotyledons and to a lesser extent in the hypocotyl, in 8-day-old seedlings, GUS staining expanded to the central cylinder, the leaf primordia and the guard cells. These results are consistent with the presence of putative root-specific and guard cell-specific *cis*-elements identified in the promoter sequence. Their additive effect on gene expression is revealed by the gradual decline in GUS staining with the progressive deletion of the respective TFBS. During reproductive development, expression was prominent in petals, anthers, filaments, pollen grains, and pistils. Progressive deletion of the promoter resulted in a gradual reduction of reporter gene expression in all flower tissues except pollen grains. GUS staining in pollen remained equally strong in all constructs aside from pVB252PAO3p, which harbors only 42 bp of the promoter. This strong GUS expression in pollen is probably accomplished by the presence of the two putative pollen-specific elements located proximal to the ORFs, indicating the significance of *AtPAO3* in pollen development and maturation. Due to the role of AtPAO3 in PA catabolism, H_2_O_2_ derived from PA oxidation might participate in signaling networks associated with microgametogenesis. In male gametophytic tissues, the generated ROS have been correlated with germline development and the activation of PCD events [58]. For example, the induced PCD of tapetal cells is essential for pollen development and the release of the microspores into the anther sacs. Moreover, Arabidopsis loss-of-function mutants of the NADPH oxidase RbohE exhibit reduced ROS levels, which result in defective pollen development due to delayed tapetal cell degeneration [59]. On the other hand, ROS have also been associated with female gametophyte patterning as well as the maintenance of the embryo sac polarity during megagametogenesis [60]. It is worth mentioning that *AtPAO3* GUS expression was also evident during pollination, in germinating pollen grains and the pollen tubes, as previously demonstrated by Fincato et al. [13]. This is in line with recent reports showing that H_2_O_2_ is a necessary component of stigma exudate, which accelerates pollen germination and ensures successful reproduction [61].

Systemic acquired resistance (SAR) triggered by pathogens and systemic acquired acclimation (SAA) triggered by abiotic stimuli such as salinity are in general mediated by the coordinated actions between ROS and other regulatory molecules such as phytohormones [40]. One of the most significant growth regulators that controls the antioxidant defense mechanisms under fluctuating environmental conditions is the phytohormone ABA [62]. Several studies have demonstrated that the application of exogenous ABA positively impacts the response of crop plants, improving their tolerance, survival, and growth potential to salinity, drought, cold, and heat [37,63]. ABA also promotes the accumulation of PAs and can interact with other hormones, such as JA and SA [49,52]. On the other hand, salinity, and drought stress lead to the production of ROS, which in turn act as key signaling molecules during ABA-mediated defense and adaptive measures, such as hypersensitive response (HR) and PCD [46]. Given this complex crosstalk signaling network, we investigated the response of the *AtPAO3* promoter under various stress conditions and hormonal signals. The in silico analysis revealed the presence of several putative TFBS, which can induce *AtPAO3* expression under salinity, osmotic stress, ABA, JA, and SA.

Salt stress involves both osmotic and ionic components. Salinity stress in plants immediately induces osmotic shock, followed by ionic stress that can lead to toxic effects due to increasing Na^+^ ions in the plant cell cytoplasm. On the other hand, the osmotic stress caused by salinity due to the reduced water uptake from the soil is also a component of the initial stages of drought stress. Thus, the component of osmotic stress represents the primary phase of both salinity and drought stress. These stresses can activate ABA-dependent and ABA-independent response pathways [63,64]. *AtPAO3* expression seems to be induced by both pathways. The promoter sequence contains both ABRE (ABA-dependent) and DRE (ABA-independent) *cis*-elements, which respond strongly to salinity, mannitol, and ABA treatments. Most of these elements are dispersed throughout the promoter sequence and exhibit an additive effect on *AtPAO3* gene expression. Deletion of one or more elements resulted in a decrease in reporter gene expression compared to the non-treated control plants. However, the inducibility of the promoter was retained in all constructs (except construct pVB252PAO3), even in those harboring only one putative *cis*-acting element. This is in agreement with the functional role of PAO genes from Arabidopsis and other plant species, and their inducibility by various stress conditions and hormonal stimuli. In *Camellia sinensis*, ABA treatment induced the expression of most *CsPAO* genes in roots and leaves and altered Spm, Put, and Spd contents, suggesting that ABA significantly influences PA biosynthesis [65]. In rice, *OsPAO3* is highly expressed in various organs and upregulated under salt stress, while overexpression of *OsPAO3* enhanced the PA content in seed coleoptiles, resulting in stronger salt tolerance at the germination stage [66]. A positive correlation between high PAO expression and salt tolerance has also been reported in rice [45]. Interestingly, however, the *A. thaliana pao1 pao5* double mutant exhibited a NaCl- and drought-tolerant phenotype by inducing genes of the salt-sensitive-, ABA-dependent, and ABA-independent pathways more strongly than wild-type upon salt treatment [44]. To maintain normal growth and development under stressful conditions, plants have also evolved complex networks that link ABA with other signaling pathways, such as JA and SA, which display both synergistic and antagonistic regulatory characteristics [48,50]. The crosstalk between these hormones helps plants detect unfavorable environmental stimuli and integrate appropriate biotic and abiotic stress responses to ensure survival. Their molecular responses include the expression of hormone-associated genes and their interactions with other growth regulators, while their physiological responses often include the activation of antioxidant systems and stomatal movements [49,51,64]. The *AtPAO3* promoter contains several putative JA- and SA-responsive cis-acting elements. The GUS reporter lines displayed increased staining after hormonal administration compared to the untreated control plants. The two JA-responsive elements seem to contribute significantly to the expression of the reporter gene under JA treatment. By deleting the most distal JA element, the expression of the reporter gene declined sharply. However, the promoter still retained its inducibility under JA treatment, and this was also maintained in the smallest functional construct harboring the most proximal JARE element. Progressive deletion of the putative SA responsive elements also resulted in a gradual decrease in GUS activity. However, the inducibility of the promoter under SA treatment was evident in all promoter fragments, also suggesting an additive effect of the SA TFBS on gene expression. On the other hand, and in line with the observed induction, deletion of a promoter fragment that lacked either a putative JA or SA element did not result in a significant decrease in reporter gene expression under the respective hormonal treatments.

Our data indicate that the transcription of *AtPAO3* is regulated by multiple environmental factors, which probably work alongside hormonal signals. Further work is needed to deeply understand PAO gene regulation and function and to unravel their full potential in plant improvement strategies.

## 4. Materials and Methods

### 4.1. Plant Material, Growth Conditions and Treatments

*Arabidopsis thaliana* (ecotype Columbia) was used in this study. Transgenic Arabidopsis plants were grown under standard conditions at 22 °C in 70% humidity with a light/dark cycle of 16h/8 h and illumination of 110 E m^−2^ s^−1^ PAR supplied by cool-white, fluorescent tungsten tubes (Osram, Munich, Germany). Selection of primary transgenic Arabidopsis plants (T1) was performed under sterile conditions on selective half strength Murashige and Skoog medium containing kanamycin (50 mg L^−1^) and cefotaxime (200 mg L^−1^). At least twenty primary transgenic plants for each construct were transferred to soil for further development. Progeny seeds from these individual lines were plated on selection plates (50 mg L^−1^ kanamycin) to obtain ten individual T2 lines. T3 seeds obtained from the ten T2 lines were again plated on selection plates to identify the T2 homozygous plants based on the segregation analysis. Seeds from 8–12 homozygous plants for each construct were used for the qualitative GUS analysis. Tissues from at least eight homozygous plants for each construct were pooled and used for protein extraction and quantitative GUS analysis. The analysis was repeated three times (three biological replicates).

Heat treatments were applied for 2 h at 37 °C on 7-day-old seedlings. For Spd treatments, plants were grown for 10 d at normal conditions and transferred thereafter to MS medium plates containing 1 mM Spd (S2626, Sigma-Aldrich, St. Louis, MO, USA). Samples were collected after 24 and 48 h for further analysis. For osmotic and salinity stress, as well as for ABA (A4906, Sigma-Aldrich), JA (J2500, Sigma-Aldrich), and SA (247588, Sigma-Aldrich) treatments, 8-day-old transgenic Arabidopsis seedlings grown under sterile conditions were transferred to 12-well tissue culture clusters containing half-strength liquid MS medium without sucrose and incubated under standard conditions on a shaking platform (150 rpm). After 24 h, the medium was removed from the wells and fresh medium was added containing 300 mM D-mannitol, 150 mM NaCl, 50 μM ABA, 50 μM JA, or 0.5 mM SA. Treatments were conducted for 6 h.

### 4.2. In Silico Promoter Analysis

The 1012 bp regulatory sequence of *AtPAO3* (gene locus *At3g59050*), including the 3′-untranslated region of the previous gene and the 5′-untranslated region of *AtPAO3*, was retrieved from TAIR (The Arabidopsis Information Resource, http://www.arabidopsis.org/ accessed on 5 June 2020). The promoter sequence was analyzed in silico using PLACE software (http://www.dna.affrc.go.jp/PLACE/ accessed on 8 June 2020) and NSITEM-PL software available at Softberry (http://www.softberry.com/berry.phtml accessed on 10 June 2020).

### 4.3. Construction of Vectors for Plant Transformation

*Arabidopsis thaliana* genomic DNA was extracted from seedlings or leaf tissues using the NucleoSpin^®^Plant II Plant Kit, according to the manufacturer’s instructions (Macherey Nagel, Düren, Germany). The DNA was subsequently used to amplify by PCR the *AtPAO3* promoter fragments of 252 bp, 479 bp, 625 bp, 765 bp, and 1012 bp upstream of the translation initiation codon, using primer pairs AtPAO3pdaR1-Bam/AtPAO3pdaF5-Sal, AtPAO3pdaR1-Bam/AtPAO3pdaF4-Sal, AtPAO3pdaR1-Bam/AtPAO3pdaF3-Sal, AtPAO3pdaR1-Bam/AtPAO3pdaF2-Sal, and AtPAO3pdaR1-Bam/AtPAO3pdaF1-Sal, respectively. The Phusion^®^ High-Fidelity DNA Polymerase (New England Biolabs, Beverly, MA, USA) was used for all PCR reactions, according to the manufacturer’s protocol. All PCR products were separated by electrophoresis on 1% agarose gels and visualized under UV light after staining with ethidium bromide (100 μg L^−1^). The various promoter amplicons of 252 bp, 479 bp, 625 bp, 765 bp, and 1012 bp were gel purified and cloned as SalI/BamHI fragments into the respective sites of linearized pBI101 binary vector ahead of the beta-glucuronidase (*GUS*) gene, generating plasmids pVB252PAO3p, pVB479PAO3p, pVB625PAO3p, pVB765PAO3p, and pVB1012PAO3p, respectively. The cloning integrity of all constructs was confirmed by DNA sequencing and restriction enzyme analysis. The primer sequences used in this study are listed in Abbreviations.

### 4.4. Plant Transformation

*Agrobacterium tumefaciens* strain GV3101 competent cells were transformed with the aforementioned vectors by using the general freeze–thaw method, as described by An et al., 1988. The transformed bacteria were then used for the stable transformation of *Arabidopsis thaliana* (Col-0) plants via the floral dip method [67].

### 4.5. Histochemical GUS Assays and Microscopy

Histochemical staining for GUS activity was performed on transgenic plants, using 5-bromo-4-chloro-3-indolyl-β-D-glucuronic acid (X-GlcA) as a substrate. Tissues were stained for 8h at 37 °C in X-GlcA reaction buffer (50 mM sodium phosphate buffer, pH 7.2, 0.5 mM potassium ferrocyanide, 0.5 mM potassium ferricyanide, and 2 mM X-GlcA), dehydrated by a series of ethanol washes, and kept in 3.7% (*w*/*v*) formaldehyde, 50% (*w*/*v*) ethanol, and 5% (*w*/*v*) acetic acid at 4 °C. Before being subjected to microscopy, tissues were treated with the clearing agent chloral hydrate (2.5 g chloral hydrate dissolved in 1 mL 60% glycerol). All samples were observed and photographed using the Zeiss Stemi 2000C stereomicroscope, equipped with the Jonoptic ProGres3 digital camera (Jenoptic GRYPHAX 2.2 software, Jena, Germany) or the differential interference contrast microscope Olympus BX50, equipped with an Olympus DP71 camera using Cell^A software (Olympus Soft Imaging Solutions, Münster, Germany). Images were processed and analyzed using Adobe Photoshop CC software (Adobe Systems Inc., CA, USA).

### 4.6. GUS Quantitative Assay

Quantitative analysis of GUS activity in treated and non-treated seedlings and plant organs was performed according to Jefferson et al. [68] with some modifications. In brief, 100 mg of tissues from control and treated samples were homogenized in 100 μL of GUS extraction buffer (50 mM sodium phosphate buffer, pH 7.0, 40 mM 2-mercaptoethanol and 10 mM Na_2_EDTA) and centrifuged at 13,000 rpm for 5 min at 4 °C. The supernatant was transferred to a new sterile tube, and 50 μL was added to a tube containing 450 μL of GUS extraction buffer with 1 mM 4-methylumbelliferyl-d-glycuronide (MUG) (Sigma-Aldrich, St. Louis, MO, USA) to initiate the enzymatic activity reaction. GUS activity was measured by monitoring the cleavage of the MUG substrate at regular time intervals and converted to pmoles 4-MU using standard curves prepared with 4-MU (Sigma-Aldrich, St. Louis, MO, USA). Fluorescence was measured with an LS50B PerkinElmer luminescence spectrometer. All measurements were repeated three times with three biological replicates of 8–12 independently transformed plants from each construct. Statistical analysis was performed using one-way or two-way ANOVA followed by Tukey’s test.

## 5. Conclusions

Herein, we provide the first comprehensive analysis of the *AtPAO3* promoter. By using a GUS reporter-aided promoter fusion approach, we generated several transgenic lines harboring progressive 5′ end deletion constructs and examined their transcriptional regulation during normal growth and development as well as in various inductive environments. Our analyses revealed that the upstream open reading frame (uORF) of *AtPAO3* and the integrity of the inter-cistronic sequence are crucial for the regulation of gene expression, while the minimal functional promoter size is 269 bp. Furthermore, the promoter sequence contains several putative TFBS, which regulate the induction and tissue-specific expression of *AtPAO3* during normal development and under salinity, drought, abscisic acid (ABA), jasmonic acid (JA), and salicylic acid (SA) treatment. Our data shed light on the fine-tuning mechanisms of *PAO* regulation and may provide the fundaments for their exploitation in breeding and plant improvement strategies.

## Data Availability

All data supporting the findings of this study are available within the paper and within its Supplementary Materials published online.

## Supplementary Materials

The following supporting information can be downloaded at: https://www.mdpi.com/article/10.3390/ijms24032317/s1.
