# Supplementary material for: GUS Reporter-Aided Promoter Deletion Analysis of A. thaliana POLYAMINE OXIDASE 3
Source: Int J Mol Sci. 2023 Jan 24;24(3):2317. doi: 10.3390/ijms24032317 (PMC9916862; doi:10.3390/ijms24032317)
Supplement: Supplementary file 1 [file ijms-24-02317-s001.zip › ijms-2150866-supplementary.pdf]

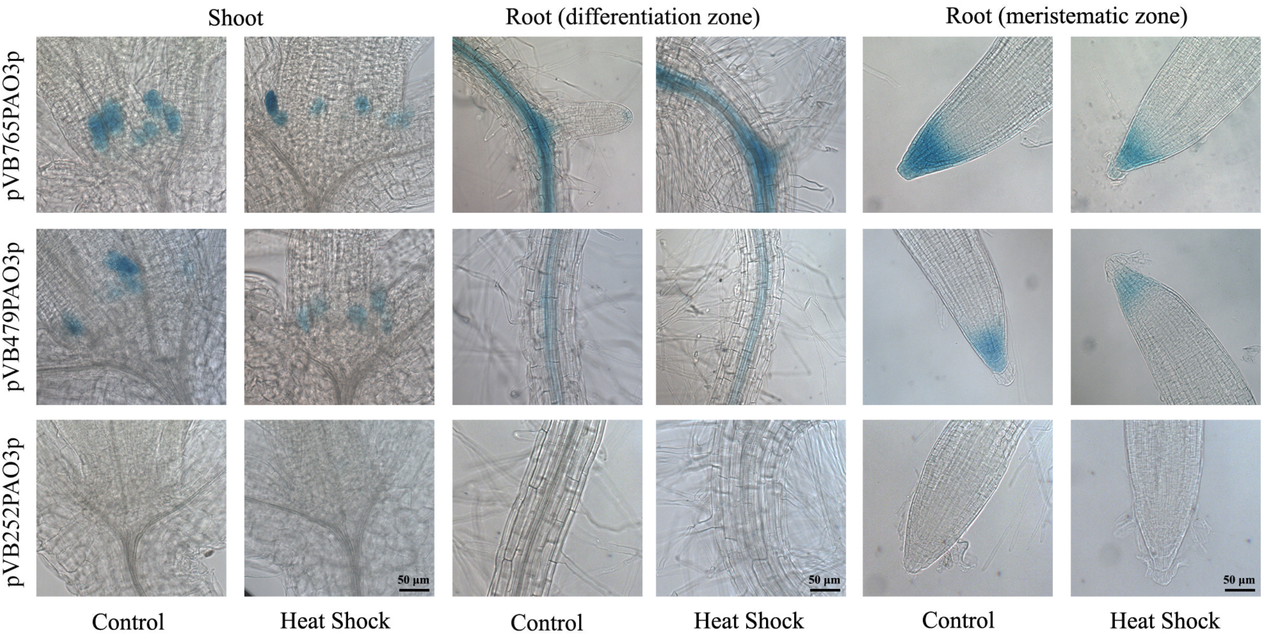

Figure S1. GUS-mediated *PAO3* expression pattern during heat stress. Representative images of histochemical GUS-stained transgenic plants harboring the constructs pVB765PAO3p, pVB479PAO3p and pVB252PAO3p. Shoot and root tissues (differentiation and meristematic zones) of control and heat treated 8-day-old seedlings showed similar expression levels, while root tips displayed a slight decrease in GUS staining.

Supplementary Table S1: Primers used in this study.

Primer name	Sequence
AtPAO3pdaF1-Sal	ATAGTCGACGGCATATAGTTTTCCTCCGA
AtPAO3pdaF2-Sal	ATAGTCGACAGGATAGACTAGGCCTGCAT
AtPAO3pdaF3-Sal	ATAGTCGACGTACGTAACGACTAGCGAGT
AtPAO3pdaF4-Sal	ATAGTCGACGTAGGTTTCCGATTAAAGCGA
AtPAO3pdaF5-Sal	ATAGTCGACTTCGTTGATCTCTGTGGAAG
AtPAO3pdaR1-Bam	ATAGGATCCGATTCAACAATGAACGGAGT

Table S2: Putative TFBs identified within the regulatory sequence of the *AtPAO3* gene locus

**Category**	**Motif**	**Consensus Sequence**	**Function**	**Position**
**ABA, Dehydration and Salinity responsive**	MYBCORE	CNGTTR	Binding site for ATMYB1 and ATMYB2. ATMYB2 is involved in regulation of genes that are responsive to water stress in Arabidopsis.	-973, -863
	MYCCONSENSUSAT	CANNTG	MYC recognition site found in the promoters of the dehydration-responsive gene rd22 and many other genes in Arabidopsis. Binding site of ATMYC2 (previously known as rd22BP1)	-973, -919, -863, -842, -816, -743, -526
	MYCATERD1	CATGTG	MYC recognition sequence necessary for expression of erd1 (early responsive to dehydration) in dehydrated Arabidopsis.	-816
	MYB2CONSENSUSAT	YAACKG	MYB recognition site found in the promoters of the dehydration-responsive gene rd22 and many other genes in Arabidopsis.	-566
	GT1GMSCAM4	GAAAAA	"GT-1 motif" found in the promoter of soybean (Glycine max) CaM isoform, SCaM-4; Plays a role in pathogen- and salt-induced SCaM-4 gene expression.	-776, -449, -344
	WRKY71OS (WRKY TF interacts with ABAR which binds ABA)	TGAC	"A core of TGAC-containing W-box" of, e.g., Amy32b promoter; Binding site of rice WRKY71, a transcriptional repressor of the gibberellin signaling pathway. WRKYs are positive and negative regulators of abscisic acid signaling in aleurone cells.	-740, -529
	DPBFCOREDCDC3	ACACNNG	A novel class of bZIP transcription factors, DPBF-1 and 2 (Dc3 promoter-binding factor-1 and 2) binding core sequence; Found in the carrot (D.c.) Dc3 gene promoter; Dc3 expression is normally embryo-specific, and can be induced by ABA.	-527
	ABRE	ACGTG	ABA responsive elements	-643, -523
	DRE	A/GCCGAC/G or TACCGACAT	Dehydration responsive elements	-549, -331

**Cell type/organ specific expression**	GTGANTG10	GTGA	"GTGA motif" found in the promoter of the tobacco late pollen gene g10.	-969, -563, -530, -415
	TAAAGSTKST1	TAAAG	Target site for trans-acting StDof1 protein controlling guard cell-specific gene expression.	-828, -533, -462
	ROOTMOTIFTAPOX1	ATATT	Root specific expression	-800, -692
	SITEIIATCYTC	TGGGCY	"Site II element" found in the promoter regions of cytochrome genes (Cytc-1, Cytc-2) in Arabidopsis; Involvement of TCP-domain RT protein-binding elements in anther- and meristem-specific RT expression of the Cytc-1 gene.	-729
	POLLEN1LELAT52	AGAAA	Pollen specific expression.	-778, -451, -346, -229, -216, -194
**Phytohormone responsive**	GT1CONSENSUS	GRWAAW	Consensus GT-1 binding site. Binding of GT-1-like factors to the PR-1a promoter influences the level of SA-inducible gene expression.	-776, -654, -449, -344
	WBOXATNPR1	TTGAC	"W-box" found in promoter of Arabidopsis thaliana (A.t.) NPR1 gene. They were recognized specifically by salicylic acid (SA)-induced WRKY DNA binding proteins.	-741
	T/GBOXATPIN2	AACGTG	"T/G-box" involves in jasmonate (JA) induction; bHLH-Leu zipper JAMYC2 and JAMYC10 proteins specifically recognize this motif.	-644
**Conserved promoter motifs**	CAATBOX1	CCAAT	CAAT-box	-397
	TATABOX3	TATTAAT	TATA-box	-351
**Others**	LTRE1HVBLT49	CCGAAA	"LTRE-1" (low-temperature-responsive element) in barley.	-991
	ANAERO1CONSENSUS	AAACAAA	One of 16 motifs found in silico in promoters of 13 anaerobic genes involved in the fermentative pathway	-772, -364

ACGTATERD1	ACGT	ACGT sequence required for etiolation-induced expression of erd1 (early responsive to dehydration) in Arabidopsis.	-672, -643, -619, -527, -426
ABRERATCAL	MACGYGB	"ABRE-related sequence" or "Repeated sequence motifs" identified in the upstream regions of 162 $Ca^{(2+)}$-responsive upregulated genes.	-644, -525
ABRELATERD1	ACGTG	ABRE-like sequence required for etiolation-induced expression of erd1 (early responsive to dehydration) in Arabidopsis.	-643, -526
CURECORECR	GTAC	GTAC is the core of a CuRE (copper-response element) found in Cyc6 and Cpx1 genes in Chlamydomonas	-621
GADOWNAT	ACGTGTC	Sequence present in 24 genes in the GA-down regulated d1 cluster (106 genes) found in Arabidopsis seed germination; This motif is similar to ABRE.	-522
CCAATBOX1	CCAAT	Common sequence found in the 5'-non-coding regions of eukaryotic genes; "CCAAT box" found in the promoter of heat shock protein genes; Located immediately upstream from the most distal HSE of the promoter; "CCAAT box" act cooperatively with HSEs to increase the hs promoter activity.	-397
AACACOREOSGLUB1	AACAAAC	Core of AACA motifs found in rice (O.s.) glutelin genes, involved in controlling the endosperm-specific expression.	-363
CANBNNAPA	CNAACAC	Core of "(CA)n element" in storage protein genes in Brasica napus (B.n.); embryo- and endosperm-specific transcription of napin (storage protein) gene, napA; seed specificity; activator and repressor.	-361
SURE1STPAT21	AATAGAAAA	Sucrose Responsive Element (SURE); A motif conserved among genes regulated by sucrose.	-345
LTRECOREATCOR15	CCGAC	Core of low temperature responsive element (LTRE) of cor15a gene in Arabidopsis (A.t.); A portion of repeat-C (C-repeat), TGGCCGAC, which is repeated twice in cor15a promoter (Baker et al., 1994); ABA responsiveness.	-331
